# The porcine respiratory microbiome: recent insights and future challenges

**DOI:** 10.1186/s42523-020-00070-4

**Published:** 2021-01-08

**Authors:** Mattia Pirolo, Carmen Espinosa-Gongora, Debby Bogaert, Luca Guardabassi

**Affiliations:** 1 Department of Veterinary and Animal Sciences, University of Copenhagen, Frederiksberg, Denmark; 2 Department of Science, Roma Tre University, Rome, Italy; 3 Center for Inflammation Research, University of Edinburgh, Edinburgh, UK; 4 Department of Pathobiology & Population Sciences, Royal Veterinary College, United Kingdom, Hawkhead Lane, North Mymms, Hatfield, Herts AL9 7TA UK

**Keywords:** Pig, Microbiota, Respiratory tract, Health, Disease, Porcine respiratory disease complex

## Abstract

Understanding the structure of the respiratory microbiome and its complex interactions with opportunistic pathogenic bacteria has become a topic of great scientific and economic interest in livestock production, given the severe consequences of respiratory disease on animal health and welfare. The present review focuses on the microbial structures of the porcine upper and lower airways, and the factors that influence microbiome development and onset of respiratory disease. Following a literature search on PubMed and Scopus, 21 articles were selected based on defined exclusion criteria (20 studies performed by 16S rRNA gene sequencing and one by shotgun metagenomics). Analysis of the selected literature indicated that the microbial structure of the upper respiratory tract undergoes a remarkable evolution after birth and tends to stabilise around weaning. Antimicrobial treatment, gaseous ammonia concentration, diet and floor type are amongst the recognized environmental factors influencing microbiome structure. The predominant phyla of the upper respiratory tract are *Proteobacteria* and *Firmicutes* with significant differences at the genus level between the nasal and the oropharyngeal cavity. Only five studies investigated the lower respiratory tract and their results diverged in relation to the relative abundance of these two phyla and even more in the composition of the lung microbiome at the genus level, likely because of methodological differences. Reduced diversity and imbalanced microbial composition are associated with an increased risk of respiratory disease. However, most studies presented methodological pitfalls concerning specimen collection, sequencing target and depth, and lack of quality control. Standardization of sampling and sequencing procedures would contribute to a better understanding of the structure of the microbiota inhabiting the lower respiratory tract and its relationship with pig health and disease.

## Background

The development of culture-independent approaches has helped to overcome the limitations of microbial cultivation, unveiling a completely new (micro) universe. Compared to culture-based approaches, metagenomic analysis, i.e. the examination of genomes and genes present in a host or environmental sample, provides a deeper understanding of microbial composition and function. Nowadays, it is possible to appreciate the diversity of bacterial communities thanks to the availability of high-throughput next-generation sequencing (NGS) methods and new advancements in bioinformatics. Sequencing of one or more of the hypervariable regions of the 16S rRNA gene (targeted NGS analysis), and shotgun sequencing of the total DNA present in a sample (untargeted NGS analysis) are the two main methods for microbiome research [1]. Early microbiome studies mostly focused on bacterial composition in the gastrointestinal tract and its influence on host health and disease [2–4]. Recent studies investigating the bacterial topography of the respiratory tract in humans [5] and animals [6] have revealed high microbial richness and diversity in the upper respiratory tract (URT) and surprisingly even in the lower respiratory tract (LRT), upending the historical notion that lungs are sterile. Consequently, there is growing interest in determining the role of the respiratory microbiome in health and disease.

Porcine Respiratory Disease Complex (PRDC) is a multi-factorial, polymicrobial respiratory disease that is generally trigged by stress factors associated with intensive farm production (e.g. overcrowding, group mixing, improper ventilation and overheating or chilling temperature) and viral pathogens, such as swine influenza virus (swIAV), porcine reproductive and respiratory syndrome virus (PRRSV) and porcine circovirus type 2 (PCV2) [7]. This disease complex is a major cause of morbidity and mortality in pig production [8] resulting in significant economic losses, treatment costs, increased antimicrobial consumption, reduced growth rates and low feed conversion efficiency [9]. The prevalence of lung lesions in slaughter pigs has been estimated to be as high as 60–65%, indicating that roughly two out of three pigs in the European Union food chain are affected by PRDC [8]. In the United States, PRDC accounted for the majority of all nursery and grower/finisher pig deaths (47.3 and 75.1%, respectively) in 2012 [10]. The bacterial species involved in this disease complex are traditionally distinguished between primary (e.g. *Mycoplasma hyopneumoniae*, *Actinobacillus pleuropneumoniae* and *Bordetella bronchiseptica)* and secondary pathogens (e.g. *Pasteurella multocida*, *Haemophilus parasuis* and *Streptococcus suis*) [11]. As some of these species are also normal commensals of the URT in healthy pigs though simultaneously cause enzootic pneumonia worldwide [12], understanding the complex interactions between the microbiome of the URT and LRT has become a topic of great scientific interest with potential impact on animal welfare and farm economy.

The role of the porcine gastrointestinal microbiome in respiratory disease has been previously reviewed by Niederwerder (2017), with particular emphasis on how the gastrointestinal microbiome impacts pig development and influences respiratory disease onset [13]. Here, we reviewed the current knowledge of the porcine respiratory microbiome and provided updated studies investigating its composition and development at different sites. Since little information is available on the role of viruses, phages, and fungi in the porcine respiratory microbiome, the goal of this review was to provide an overview of the bacterial topography of the healthy pig respiratory tract and how changes in the microbial community composition may be positively or negatively correlated to disease occurrence based on the current scientific evidence. At the same time, we identified and discussed methodological pitfalls of respiratory microbiome studies with reference to this animal species.

## Literature search

**Fig. 1:**
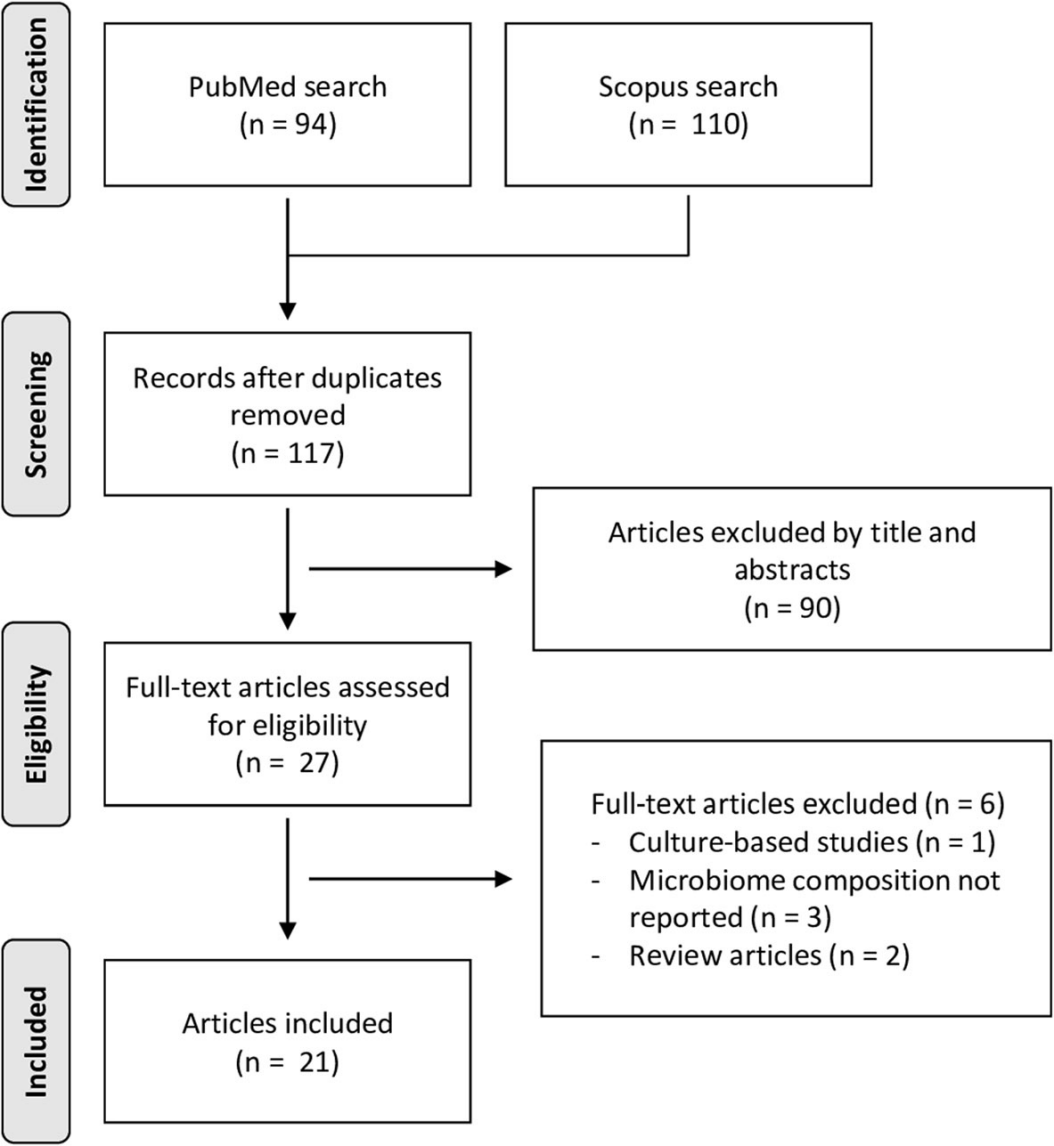
Flowchart of the literature search and studies selection

A literature search in the PubMed and Scopus databases was started on August 1, 2019 and ended on September 30, 2020. Relevant peer-reviewed publications were searched applying the following search criterion: (porcine OR pig OR swine) AND (microbiome OR microbiota) AND (pulmonary OR lung OR respiratory OR nasal OR oropharyngeal OR tonsil). A flow chart illustrating the selection strategy is shown in Fig. 1. Four exclusion criteria were applied: *i*) studies where the microbiome composition does not refer to the respiratory tract, *ii*) studies using exclusively culture-based methods, *iii*) studies in languages other than English, and *iv*) reviews.

Out of 204 records identified through database searching (94 in PubMed and 110 in Scopus), 117 non-duplicate articles were screened, and 21 eligible papers were selected based on the exclusion criteria. The main features of the eligible studies are summarized in Table 1. All but one article (95.2%) included 16S-based metataxonomic data, with the remaining study (4.8%) evaluating the microbiome composition through shotgun metagenomics. The microbiota composition of the URT was assessed in 16 studies (76.2%). Only five studies (23.8%) focused on the LRT microbiome.

**Table 1:** Summary of studies on the porcine respiratory microbiota

Study population		Sampling		Positive and negative sequencing controls	Sequencing platform (16S rRNA gene region)	Data processing software (reference database)	Main finding	Reference
No. of animals	Age of animals	Respiratory tract	Sample type					
**Microbiome development**								
10 Yorkshire-Landrace pigs	0–7 weeks	URT	Nasal swab	Absent	Illumina MiSeq (V4)	mothur (RDP)	The nasal microbiota of pigs undergoes a remarkable evolution during the first 7 weeks of life	21
34 piglets	0–4 weeks	URT	Tonsil swab	Extraction control, *Escherichia coli* DH5α genomic control and mock community DNA	Illumina MiSeq (V4)	mothur (SILVA)	The tonsillar microbiome of piglets does not show composition stability until 3 weeks, and shared similarities with the sow vaginal and teat skin microbiome	18
16 piglets	0–19 weeks	URT	Tonsil swab	Extraction control	Illumina MiSeq (V4)	mothur (SILVA)	Significant community shifts during the development of the tonsillar microbiome are correlated with disruption events, including weaning, changes in feed, antibiotic treatment, and movement to new housing	23
**Bacterial topography in healthy animals**								
8 pigs from a herd with health status and 4 pigs from a healthy herd with a history of chronic respiratory problems	18–20 weeks	URT	Tonsil swab and tissue	Absent	GS 454 FLX-Titanium (full-length 16S rRNA gene)	RDP pyrosequencing pipeline (RDP)	*Actinobacillus*, *Pasteurella* and *Haemophilus* form the tonsillar core microbiome	38
5 MRSA-positive and 8 MRSA-negative pigs from a farm that used a liquid feeding system and 7 MRSA-negative pigs from a farm that used conventional feeding practices	1–2 weeks prior to slaughter	URT	Nasal swab	Absent	Illumina MiSeq (V4)	mothur (Silva)	The nasal cavity of slaughter-age pigs harbours a rich and diverse microbial community that can be influenced by diet and farm management practices	24
100 pigs classified as *S. aureus* carriers (*n* = 44) and non-carriers (*n* = 56)	Last 3 week of production cycle	URT	Nasal swab	Absent	GS 454 FLX Titanium (V3-V5)	BION-meta (RDP)	The nasal microbiota may play a role in the individual predisposition to *S. aureus* nasal carriage in pigs	36
6 pigs	Not specified	URT	Swabs and biopsies of true and false vocal folds of larynges	Extraction and PCR controls	GS 454 FLX-Titanium (V3-V5)	mothur (SILVA)	There is no difference in laryngeal microbial communities when sampled via swab or biopsy from either the true or false vocal fold	42
Two lavage pools from 20 lungs presenting enzootic pneumonia signs and 20 lungs without macroscopic pneumonia signs	Not specified	LRT	BAL	Absent	GS 454 FLX Titanium (shotgun metagenomics)	MG-RAST (M5NR)	In farms with a history of chronic respiratory problems, *Mycoplasma hyopneumoniae* is present in lungs with or without enzootic pneumonia signs	46
20 lungs from the six-generation offspring of a population crossed by eight pig breeds (seven healthy, six and seven with moderate or severe lesion, respectively)	240 ± 3 days	LRT	BAL	Extraction and PCR controls	Illumina MiSeq (V3-V4)	QIIME (Greengenes)	Reduced microbial diversity but more biomass was observed in severe-lesion lungs compared to healthy lungs	43
20 Duroc–Landrace–Yorkshire piglets (10 PRRSV-challenged and 10 controls)	8–10 weeks	LRT	BAL	Absent	Illumina MiSeq (V3-V4)	mothur (SILVA)	Challenging pigs with the PRRSV increase the presence in the lungs of opportunistic pathogens, including *Haemophilus parasuis* and *Mycoplasma hyorhinis*	45
**Microbiome relationship with disease and productivity**								
100 piglets from 4 farms with Glässer’s disease outbreaks and from 6 control farms (10 piglets/farms)	3–4 weeks before weaning	URT	Nasal swab	Absent	Illumina MiSeq (V3-V4)	QIIME (Greengenes)	The nasal microbiota of piglets is associated to the clinical status of the farm, leading to different susceptibilities to invasive infection by opportunistic pathogens	35
30 pigs	Not specified	URT	Nasal swab	Extraction control, PCR control and *Staphylococcus* mock community	Illumina MiSeq (V1-V2)	BION-meta (RDP)	The MRSA nasal colonization is negatively correlated with the level of *Streptococcus*	37
33 piglets (23 diseased and 10 healthy)	2–3 weeks	URT	Oropharyngeal swab	Extraction control	Illumina MiSeq (V3-V4)	RDP Classifier (SILVA)	*Moraxella*, *Veillonella*, and *Porphyromonas* may play a potential role in PRDC and *Lactobacillus* may have a protective role against respiratory diseases	41
0 piglets	8 weeks	URT	Nasal swab	Absent	Illumina MiSeq (V1-V3)	QIIME (Greengenes)	The impact of parenteral antibiotics on the pig nasal microbiota is variable and modulates the nasal microbiota structure	29
31 piglets (based on the BioSample number in NCBI Sequence Read Archive)	3/4-weeks	URT	Nasal swab	Absent	Illumina MiSeq (V3-V4)	QIIME (RDP)	Removal of perinatal antimicrobials at weaning increases the bacterial diversity in nasal microbiota and the relative abundance of beneficial genera	31
44 piglets	6 weeks	URT	Tonsil surface	Extraction control and mock community	Illumina MiSeq (V4)	DADA2 (SILVA)	Chlortetracycline administration enhances the shedding and colonization of multidrug resistant *S. Typhimurium* in pigs	39
65 piglets	2–3 weeks	URT	Nasal swab and tonsils tissue	Absent	Illumina MiSeq (V4)	mothur (SILVA)	Short-term exposure to broad-spectrum antibiotics (oxytetracycline) can disturb the URT microbiota	30
120 Duroc–Landrace–Yorkshire pigs (the nasal microbiota of 72 pigs was analysed)	Not specified	URT	Nasal swab	Absent	Illumina MiSeq (V3-V4)	QIIME (Greengenes)	Concentrations of ammonia higher than 25 ppm may cause respiratory damage and even pneumonia by affecting the colonization rates of harmful bacteria and beneficial bacteria	26
175 piglets	19–22 days	LRT	Bronchial mucosa	Absent	Illumina MiSeq (V3-V4)	QIIME (SILVA)	Increasing the physical complexity of rearing environment provides suboptimal conditions for establishing a healthy microbial community in the growing pigs	25
8 piglets	1–7 weeks	URT	Oropharyngeal swab	Extraction control, PCR control and mock community	Illumina iSeq (V4)	QIIME (Greengenes)	The rate of average daily gain of piglets is associated with a characteristic oropharyngeal microbial signature	56
28 Duroc–Landrace–Yorkshire pigs (eight healthy and 20 PRDC-affected)	270 ± 3 days	LRT	BAL	Absent	Illumina MiSeq (V3-V4)	QIIME (RDP)	In PRDC-affected pigs, the proportion of harmful genera increased, and the richness of beneficial genera decreased	44

*Abbreviations*: *BAL* Bronchoalveolar lavage, *LRT* Lower respiratory tract, *PRDC* Porcine respiratory disease complex, *PRRSV* Porcine reproductive and respiratory syndrome virus, *RPD* Ribosomal Database Project, *URT* Upper respiratory tract

## Initial colonization and development of the respiratory tract microbiome

The pig respiratory tract can be divided into two parts, the URT, consisting of the nose, pharynx, larynx, and the LRT, which comprises the trachea and lungs [14]. The anatomical development of the structures of the porcine respiratory tract is a complex multistage process that begins at the embryonal stage, with the formation of the olfactory placodes, the laryngotracheal groove and the lung buds, and continues after birth, with the ventral scroll bone development and the progression of alveolar formation [15]. The origin of the first bacteria colonizing the airways in new-born piglets remains unclear. According to the sterile womb paradigm in humans, bacteria are acquired from both the mother (vertical acquisition) and the environment (horizontal acquisition) during and shortly after birth [16]. However, airway colonization might begin in utero, likely during intraamniotic infection (chorioamnionitis), though studies are needed to confirm this hypothesis in pigs. Regardless of when microbial colonization starts, either in utero or postnatally, all external and internal surfaces of new-borns become heavily colonized afterbirth. Wang et al. (2013) showed that microbial development of the piglet nasal microbiota is strongly shaped by external factors, such as delivery mode and feeding type [17].

The tonsillar microbiota of piglets within the first 8 hours after birth resembles the sow vaginal and teat skin microbiota, indicating that these two body sites represent an important source for early colonization of the URT [18]. Similarly, the bacterial species detectable in the URT of infants during the first few hours of life resemble those occurring in the mother’s vaginal or skin microbiota depending on whether the child is born by natural birth or C-section, respectively [19, 20]. A longitudinal study by Slifierz et al. (2015) showed remarkable changes in the composition and structure of the nasal microbiota of piglets during the first 7 weeks after birth, which tended to stabilise after two-to-three weeks post-weaning [21]. Another study indicated that the microbial communities of tonsils of healthy piglets initially clustered by litter, but then converged by 3 weeks of age, regardless of litter or housing [18]. While the influences of environmental exposures on the respiratory microbiome composition in pigs are generally known, the degree to which host genetics plays a role in structuring the airway microbial community is less well understood.

Weaning has a pivotal influence on the early microbiome composition of piglets [13]. Major microbial shifts occur in response to the transition to solid-feed in the gut microbiome of healthy suckling piglets [22]. This holds true also for the URT microbiome, as weaning has been associated with an increase in the relative abundance of *Streptococcaceae* and a decrease of *Moraxellaceae* abundance; this might be result of changes in feed, but also in housing conditions and antimicrobial exposure [18, 23]. Feeding strategies and the flooring system have been proposed to impact the composition of the URT respiratory microbiome [24, 25]. Significant differences in the relative abundance of some genera, including *Macrococcus*, *Pseudomonas*, *Corynebacterium* and *Fibrobacter*, have been reported in the nasal microbiota of liquid-fed pigs compared with conventional diet fed pigs [24]. Simple-slatted systems were proposed by Megahet et al. (2019) to provide better conditions for the establishment and development of a balanced respiratory and intestinal microbiota compared to straw-based rearing systems. The authors hypothesized that harmful taxa could prevail in complex environments and might decrease the opportunity for beneficial bacteria to flourish, which may impede the amplification of microbial diversity [25].

Ammonia emissions are known for their potential negative consequences on farming environments, the ecosystem, and human and animal health [26]. The concentration of gaseous ammonia in the farm environment has been shown to affect the porcine airway [27]. In vivo testing showed that an increase in ammonia levels, especially above 25 ppm, significantly damaged tracheal mucosa and alveoli, and decreased growth performance [26]. In addition, ammonia levels above 25 ppm reduced the colonization of beneficial bacteria such as *Lactobacillus* and increased the number of *Moraxella* and *Streptococcus* species in the nasal cavities of exposed pigs [26].

Antimicrobial exposure is another key environmental factor that can affect the development of URT microbiome at early stages of life, especially because antimicrobials are widely used for control of post-weaning diarrhoea [28]. Antimicrobial treatment perturbs the URT microbiome homeostasis regardless of the drug used (tylosin, ceftiofur, tulathromycin, oxytetracycline or penicillin) and the administration route (in-feed, parenteral or intramuscular) [29, 30]. The changes observed in the URT microbiota are however specific to each antimicrobial, in some instances leading to dramatic changes in the relative abundance of some bacterial taxa [29]. For instance, parenteral administration of ceftiofur, a broad-spectrum third-generation cephalosporin with activity against both Gram-positive and Gram-negative bacteria, was shown to increase the relative abundance of *Clostridium,* and to decrease the relative abundance of *Moraxella*, *Streptococcus* and *Neisseria* in the nasal microbiome of healthy pigs [29]. A similar pattern was observed after parenteral administration of the macrolide tulathromycin, whereas parenteral administration of oxytetracycline had a lower effect on the porcine nasal microbiome, as only minor changes in *Clostridium* and *Streptococcus* populations were observed [29]. The impact of oxytetracycline on the pig URT microbiome has also been addressed by Mou et al. (2019), who compared the tonsillar and nasal microbiota after in-feed or parenteral administration of oxytetracycline. Interestingly, oral administration resulted in a more pronounced decrease in the bacterial diversity and a relative increase of the genera *Actinobacillus* and *Streptococcus* [30]. In another study, removal of perinatal antimicrobial treatment was associated with increased bacterial diversity and relative abundance of *Prevotella* and *Lactobacillus* in the nasal microbiota at weaning [31].

No studies have examined the initial colonization and development of the LRT microbiome in pigs. Human studies indicate that bacterial colonization of lungs is the result of a dynamic equilibrium maintained by immigration (micro-aspiration of URT bacterial members and inhalation of ambient air) and elimination (cough, mucociliary clearance, and immune system activity) [5, 32]. The scenario of a self-sustaining LRT, which takes advantage of a mutualistic interrelationship with the URT, has been also proposed for cattle [33, 34]. No information is available on the relationship between the URT and LRT in pigs since all studies focused on either the URT or LRT. The following paragraph provides an overview of the current knowledge on the diversity and composition of the microbial community residing in different parts of the respiratory tract of healthy pigs.

## Bacterial topography of the healthy respiratory tract

**Fig. 2:**
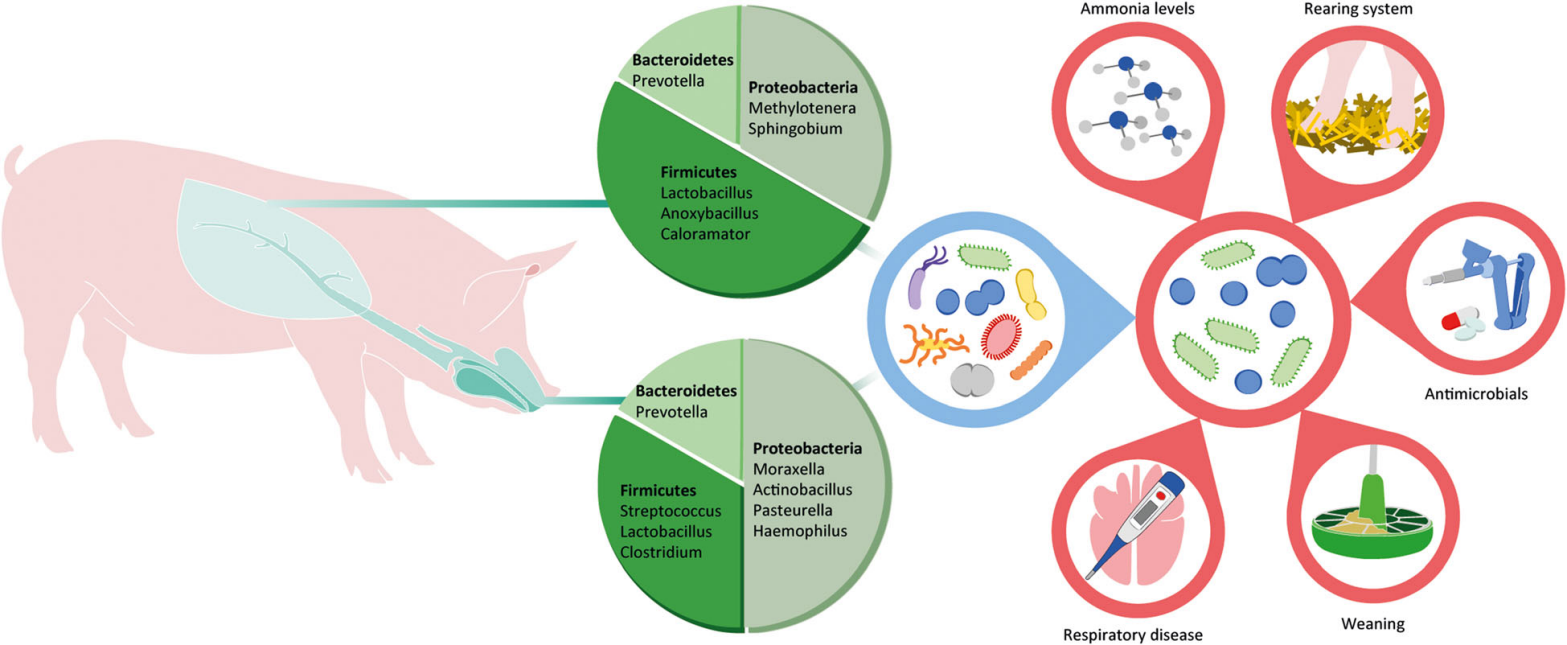
Bacterial topography and factors shaping the porcine respiratory microbiome. The porcine respiratory microbiome is divided into the URT (upper pie chart) and the LRT (lower pie chart), which are mainly colonized by members of the *Proteobacteria*, *Firmicutes*, and *Bacteroidetes*. Under physiological conditions, the respiratory tract hosts a rich and diverse microbial community (left circle). Several factors, including respiratory disease onset, farm management practices and antimicrobial treatment, contribute to reducing the bacterial diversity in the imbalance state (right circle)

*Proteobacteria* and *Firmicutes* were consistently reported as the predominant phyla colonizing the nasal cavity of healthy pigs, irrespective of their age [21, 29, 35, 36] (Fig. 2). *Bacteroidetes* and *Actinobacteria* were also identified, although at lower prevalence [21, 29, 35, 36]. At the genus level, *Moraxella* was the dominant genus more often reported in the nasal cavity of healthy pigs throughout all the phases of production cycle, followed by *Streptococcus*, *Clostridium* and *Lactobacillus* [21, 24, 29, 31, 35, 37].

The pig URT microbiota composition has also been investigated by sampling the surface and deep tissue of the tonsils [38]. At the phylum level, the composition of the tonsillar microbiome of adult pigs resembled that of the nasal microbiota, with *Proteobacteria* and *Firmicutes* as the predominant taxa [38] (Fig. 2). However, in contrast to the nasal microbiota, *Actinobacillus*, *Pasteurella* and *Haemophilus* formed the tonsillar core microbiome (e.g. the most widespread taxa that are found in all samples) at the genus level, although member of the *Veillonella* and *Peptostreptococcus* were also identified [38, 39]. Unlike the nasal cavity, *Moraxella* was reported as a less frequent colonizer of the tonsils [38], in line with human studies [40]. The oropharyngeal microbial community of healthy piglets resembled that of the tonsils, with *Streptococcus*, *Actinobacillus* and *Lactobacillus* forming the core microbiome [41]. *Streptococcus* was also reported as a common coloniser of the larynx of healthy finishing pigs [42], although discrimination at the species level between commensal and pathogenic streptococci (i.e. *S. suis*) was not possible due to lack of a deep metagenomic analysis.

We only identified four studies investigating the LRT microbiota composition in healthy pigs by 16S rRNA gene sequencing (Table 1), and their results often diverged. Two studies reported *Proteobacteria* and *Firmicutes* as the main phyla, but the third most dominant phylum was *Bacteroidetes* in the study by Huang et al. (2019) and *Tenericutes* in the study by Li et al. (2020) [43, 44]. Conversely, Jiang et al. (2019) found *Firmicutes* as the dominant phylum, whereas *Proteobacteria* and *Bacteroidetes* formed only a minor portion of the healthy lung microbiota [45]. Even bigger differences were observed at the genus level, with the predominant genera being *Methylotenera*, *Prevotella*, *Sphingobium* and *Lactobacillus* according to Huang et al. (2019), *Escherichia*, *Mycoplasma*, *Lactococcus* and *Macrococcus* according to Li et al. (2020), and *Anoxybacillus* and *Caloramator* according to Jiang et al. (2019) [43–45]. The discrepancies between the three studies could be due to age disparities in the studied animals, and to methodological differences in sampling and data analysis (Table 1). The breeding environment could also have contributed to such discrepancies, given that in one study animals were housed in separate units in a biosafety level 2 (BSL2) facility [45], whereas animals were raised under natural conditions in the other two studies [43, 44]. In addition, two studies failed to include negative and positive control samples [44, 45], and some of the genera identified (e.g. *Sphingobium* and *Escherichia*) could be potential contaminants, especially in low biomass samples (see Section Methodological study limitations).

Bacterial composition at the phylum and genus level were not reported in another study that characterized the LRT microbiome by shotgun sequencing [46]. That study reported *Mycoplasmataceae*, *Bradyrhizobiaceae* and *Flavobacteriaceae* as the most abundant families in pooled bronchoalveolar lavage (BAL) samples from healthy pig lungs, with *M. hyopneumoniae*, *Bradyrhizobium japonicum* and *Flavobacterium johnsoniae* as the predominant species. Fungal and viral communities are known interact with members of the bacterial microbiome in humans [32]. To date, the porcine airway mycobiome has not been investigated, whereas the virome residing in the porcine airways has been evaluated by only two studies focused on the URT [47, 48]. Results of both studies converge towards a complexity of viral communities in the pig upper airways [47, 48].

## Relationship of the respiratory microbiome with disease

Changes in microbial community composition and alteration to the abundance and diversity of the airway microbiome are associated with the impairment of the host’s health [35, 49, 50]. The loss of a stable ecosystem (homeostatic healthy state), which is resistant to colonization by pathogens, favours the progression towards an unstable community (i.e. dysbiosis) that is predisposed to infection and inflammation (pre-disease state) [51]. The decline towards the pre-disease state in the respiratory system, is often accompanied by unfavourable events, including partial loss of mucosal barrier, colonization by opportunistic pathogens and release of proinflammatory cytokines, which ultimately lead to the disease state [51].

The relationship between the respiratory microbiota and PRDC was evaluated by two recent studies comparing healthy and diseased pigs [41, 44]. Genera that include common bacterial PRDC pathogens, namely *Streptococcus*, *Haemophilus*, *Pasteurella*, and *Bordetella*, were found to be more relatively abundant in BAL samples from diseased animals, often in combination with a reduction of beneficial genera (e.g. *Lactococcus* and *Lactobacillus*) [44]. PRDC is also associated with changes in the composition of the URT microbiota, as evidenced by the higher abundance of *Moraxella*, *Veillonella*, and *Porphyromonas* in the oropharynx of PRDC-affected pigs [41]. As observed in human patients with respiratory disease [52–54], low species richness and diversity in the URT was associated with piglets affected by Glässer’s disease [35], a systemic disease often associated with pneumonia. Metataxonomic profiling of the microbiome of porcine lungs with and without macroscopic lesions revealed that microbial diversity was lower in porcine lungs with severe lesions than in healthy or moderately affected lungs [43]. At the phylum level, a significant reduction in the relative abundance of *Firmicutes* and *Actinobacteria* was observed in lungs with severe lesions compared to healthy lungs, which was associated with an increase of *Mycoplasma* and a reduction of *Lactobacillus* and *Streptococcus* at the genus level [43].

The porcine URT microbiome has been widely investigated with a focus on the spread of methicillin-resistant *Staphylococcus aureus* (MRSA), an emerging zoonotic pathogen in pig farming [55]. In a longitudinal study of *S. aureus* carriage in Danish pig farms, 20 taxa were associated with non-carriage of *S. aureus* in the nasal microbiome, including species from the *Leuconostocaceae* and *Lachnospiraceae* families [36]. In another study, MRSA colonization of the nasal cavity was inversely correlated to the occurrence of *Streptococcus spp.*, suggesting a possible antagonism between streptococci and MRSA [37]. Concerns over the increasing emergence of antibiotic-resistant bacteria have prompted efforts to identify alternatives to antimicrobials in animal production, such as probiotics. Members of the *Lactobacillus* genus have been widely proposed as probiotic agents thank to their ability to increase natural killer cell activity, reduce proinflammatory cytokine production and protect biological niches [13]. Although the effect of *Lactobacillus* spp. on the respiratory tract of pigs remains unclear, a significant reduction in the relative abundance of *Lactobacillus* was observed in both the oropharynx and lungs of PRDC-affected pigs, as compared to healthy animals [41, 43, 44]. Taken together, this evidence supports the hypothesis that *Lactobacillus* spp. could exert a protective role against respiratory pathogens.

Very little is known about the relationship of the respiratory microbiome with pig productivity. A single study suggested that the rate of average daily gain of piglets could be associated with a characteristic oropharyngeal microbial signature and that manipulating the oropharyngeal microbiota in pre-weaned piglets could influence weight gain [56]. In addition, several reports have shown the importance of the gut microbiome in pig productivity through its effects on digestion, feed efficiency and immune stimulation [57]. These studies suggest that there might be close relationship between the microbial community colonizing different body sites, including the respiratory tract, and animal’s weight gain and other productivity parameters.

## Methodological study limitations

Despite the advancement of the sequencing and bioinformatics tools, unique technical challenges and pitfalls bias the results of respiratory microbiome studies. First, sequencing target and strategy may lead to discrepancies in the abundance of some taxa [58–60]. Among these, differences in the *i*) 16S rRNA gene variable region targeted, *ii*) primers employed, *iii*) length of the reads produced by the sequencing platforms, *iv*) data processing software and *v*) reference databases are among the major methodological problem with the largest impact on the outcomes of the studies [58, 61]. Notably, the reviewed studies showed a remarkable variability in the selection of 16S rRNA region, sequencing platform and bioinformatic tools for analyses (Table 1). Therefore, standardisation of protocols for metagenomic profiling is strongly encouraged.

Specimen collection from both the URT and LRT plays a critical role in microbiome studies. Nasal and tonsil swabs are the most frequent samples examined in URT microbiome studies (Table 1). Nasal sampling has the advantages of being non-invasive and minimising distress, whereas non-invasive tonsil and larynx swabs yielded similar results with respect to tissue specimens, eliminating the need to euthanize animals to collect the tissue [38, 42]. The inherent difficulty in sampling the LRT and the remarkably low bacterial burden in healthy lungs are some of the technical challenges hampering LRT microbiome research [62, 63]. In pigs, BAL samples of healthy animals have an average bacterial load of 10^4^–10^5^ CFU/ml, although it has been reported that most of the detectable bacterial DNA in the lung parenchyma is DNase I sensitive and corresponds to dead bacteria [64]. In a study by Siqueira et al. (2017) due to difficulties in obtaining large quantities of DNA from individual lungs, the material retrieved from 20 lungs with suggestive enzootic pneumonia signs and from 20 lungs without macroscopic signs was pooled [46]. However, while pooling samples can be helpful to overcome the issues related to low DNA yield in BAL samples, it does not allow for the assessment of individual variation [65].

Although contamination of BAL samples during passage of the endoscope through the URT is unavoidable, previous studies in pigs and humans have shown that this has a negligible effect on respiratory acquired through bronchoscopy [5, 64]. Nevertheless, transbronchial BAL collection should be preferred to transoral [33]. Tracheobronchial swabbing is a less invasive alternative to BAL for LRT specimen collection that is well established in pig clinical practice, especially for detection of PRDC pathogens [66, 67]. Collection of tracheobronchial swabs does not require anaesthesia, which is necessary prior to BAL sampling. Further studies are warranted to evaluate the use of tracheobronchial swabs as proxies for lung microbiome studies.

Contrary to cattle studies, in which BAL was mainly performed by trans-tracheal aspiration on mildly sedated animals [68, 69], previous studies in pigs used BAL samples collected immediately after slaughter, with consequent risks of bacterial contamination via aspiration of scalding water. Scalding operations may affect the lung microbiome composition due to ingestion of water in scalding tanks (at 70 °C), which is usually reused and becomes contaminated by bacterial DNA from the skin of dead animals [70]. Therefore, collection of post-mortem BAL at slaughter poses serious risks of contamination.

Lastly, contamination through handling and processing of samples is considered another major pitfall in respiratory microbiome studies [71]. Most studies investigating the porcine airway microbiome failed to include positive (i.e. mock communities) and negative (i.e. extraction control and PCR blank) control samples (Table 1). This omission, paired with the investigation of relatively low biomass microbiomes, provides a source of uncertainty in the results, which are potentially indistinguishable from contaminations. This is why inclusion of negative controls for sampling, DNA extraction and sequencing, and positive controls for DNA extraction and sequencing have been proposed as standards in microbiome research [72].

## Conclusions and future perspectives

Recent technological advancements have allowed us to appreciate differences in microbiome composition between different URT sites (e.g. nasal cavity versus tonsils and oropharynx), and highlight associations between URT microbial structure and the onset of respiratory disease. As with humans and other animal species, appears that the respiratory microbiome of healthy pigs is characterized by higher microbial richness and evenness compared to diseased pigs, suggesting that an imbalance in the microbial composition plays a key role in susceptibility to PRDC. An unstable microbiota seems to enhance colonization of the URT epithelial surfaces by opportunistic pathogenic bacteria, which can further progress into the LRT causing lung disease.

Understanding the complex interactions and relationships of the pig respiratory microbiome with health and disease is however at its early stage. More research is needed to clarify the interaction between commensal microorganisms and opportunistic pathogens, including bacteria, viruses and fungi residing in the airway, and their role in the development of PRDC. As the current knowledge of the role of the microbiome in PRDC is based on comparisons of healthy and disease lungs, longitudinal studies are warranted to determine the ecological changes in the lung microbiota during disease development, and how such changes relate to antimicrobial exposure in diseased pigs. The vast majority of studies on the porcine respiratory microbiome focused on the URT bacterial community alone. Hence, further studies investigating both the URT and LRT microbial structure in pigs are warranted to clarify how and to what extent the lung microbiota overlaps with that inhabiting the upper airways. Moreover, in most studies metataxonomic profiling was resolved only at the genus level, possibly due to the choice of the 16S rRNA gene region and to incomplete 16S reference database. Therefore, data at the species level are required to magnify the structure of the respiratory microbial community in pigs and its relationship with disease.

Viral and fungal communities play a key role in the microbiome of the human and animal respiratory tract. Nevertheless, studies investigating the pig respiratory virome are scarce [47, 48], whereas to the best of our knowledge, studies of mycobiome of the porcine respiratory tract are completely lacking. Expanding the community profiling to non-bacterial members of the pig respiratory microbiome (e.g. viruses, phages, and fungi) could generate an accurate snapshot of the entire microbial community and add further insights on how these members interact with the respiratory bacterial microbiota. Such issues could be solved by shotgun sequencing, although results might be hampered by low quantities of biomass and DNA, especially in LRT samples. Finally, appropriate specimen collection, uniform laboratory practice (selection of the 16S rRNA region, sequencing platform, and data processing software) and inclusion of sequencing controls should be envisioned in future studies addressing the porcine respiratory microbiome.

## Data Availability

Not applicable.

## Abbreviations

BAL: Bronchoalveolar lavage
LRT: Lower respiratory tract
MRSA: Methicillin-resistant *Staphylococcus aureus*
PRDC: Porcine respiratory disease complex
URT: Upper respiratory tract
